# Gender difference in the association between aminotransferase levels and hypertension in a Chinese elderly population

**DOI:** 10.1097/MD.0000000000006996

**Published:** 2017-05-26

**Authors:** Lei Wu, Yao He, Bin Jiang, Miao Liu, Shanshan Yang, Yiyan Wang, Jing Zeng, Yao Yao, Jianhua Wang

**Affiliations:** a Department of Epidemiology, Institute of Geriatrics, Chinese People's Liberation Army General Hospital; b State Key Laboratory of Kidney Disease, Chinese People's Liberation Army General Hospital; c Beijing Key Laboratory of Aging and Geriatrics, Chinese People's Liberation Army General Hospital; d Department of Acupuncture, Chinese People's Liberation Army General Hospital, Beijing, China.

**Keywords:** aminotransferase, elderly population, gender-difference, hypertension

## Abstract

Few epidemiological studies have examined the association between serum aminotransferase levels and hypertension, and have yielded inconsistent results.

A cross-sectional study was performed in a Chinese rural elderly population. A total of 2174 participants with normal range of aminotransferase levels and without excessive drinking were included in the present study. Alanine aminotransferase (ALT) and aspartate aminotransferase (AST) levels were measured on fasting morning serum samples using the Kinetic method. Hypertension was defined as systolic blood pressure ≥140 mm Hg and/or diastolic blood pressure ≥90 mm Hg and/or receiving treatment for hypertension. Multiple logistic regression was used to estimate the association between gender-specific aminotransferase levels and hypertension.

Increased serum ALT but not AST level was positively associated with hypertension. After adjusting for potential confounding variables, the association of hypertension and ALT level was only significant in women: for each 1 IU/L elevation of ALT level, the adjusted odds ratio (OR), and corresponding 95% confidence interval (CI) of hypertension was 1.04 (1.01, 1.07); the ORs of hypertension increased across tertiles of ALT, and the ORs (95% CIs) were 1.00, 1.17 (0.85, 1.60), and 1.63 (1.15, 2.31 (*P* value for trend = .021). Furthermore, the association was only significant in central obesity women or nondrinking women.

ALT level was significantly associated with hypertension only in women in a Chinese rural elderly population. Further studies are warranted to explore the possible gender-related association and to extend them to different populations.

## 1 Introduction

Hypertension remains the biggest single contributor to the global burden of disease and mortality.^[1]^ Kearney et al^[2]^ reported that the number of people affected by hypertension was predicted to increase by about 60% in 2025 in almost all parts of the world. Hypertension is one of the most important treatable risk factors for stroke, many forms of coronary heart disease, cognitive decline, and dementia.^[3–5]^ Thus, it is important to identify the risk factors of hypertension and to prevent the progression of hypertension and its subsequent-related diseases.

It has been suggested that nonalcoholic fatty liver disease (NAFLD) is caused by accumulation of fat in the liver of the individuals who are not excessive alcohol users.^[6]^ Exercise habits of patients with NAFLD may benefit the parameters of metabolic syndrome.^[7]^ Serum markers of liver injury, such as gamma-glutamyltransferase (GGT), aspartate aminotransferase (AST), and alanine aminotransferase (ALT), are also increased in NAFLD patients.^[8]^ Apart from liver diseases, accumulating evidence has shown that GGT is a risk indicator for hypertension in different countries.^[9–16]^ However, few epidemiological studies have examined the association between serum aminotransferase levels and hypertension, and have yielded inconsistent results. Some researchers reported that ALT level was associated with an increased risk of elevated blood pressure,^[14–16]^ whereas other studies did not observe a significant association.^[9,10]^

The measurements of AST and ALT involve simple, inexpensive, and routine liver function tests, and thus it is of interest to establish the possible association between aminotransferase levels and hypertension. If the association exists, it can help to monitor the risk of hypertension in the routine test with low cost. Therefore, we conducted a cross-sectional study to investigate the association of aminotransferase (AST and ALT) levels with hypertension and to explore its possible gender difference in Chinese rural elderly participants.

## 2 Design and methods

### 2.1 Study design

**Figure 1:**
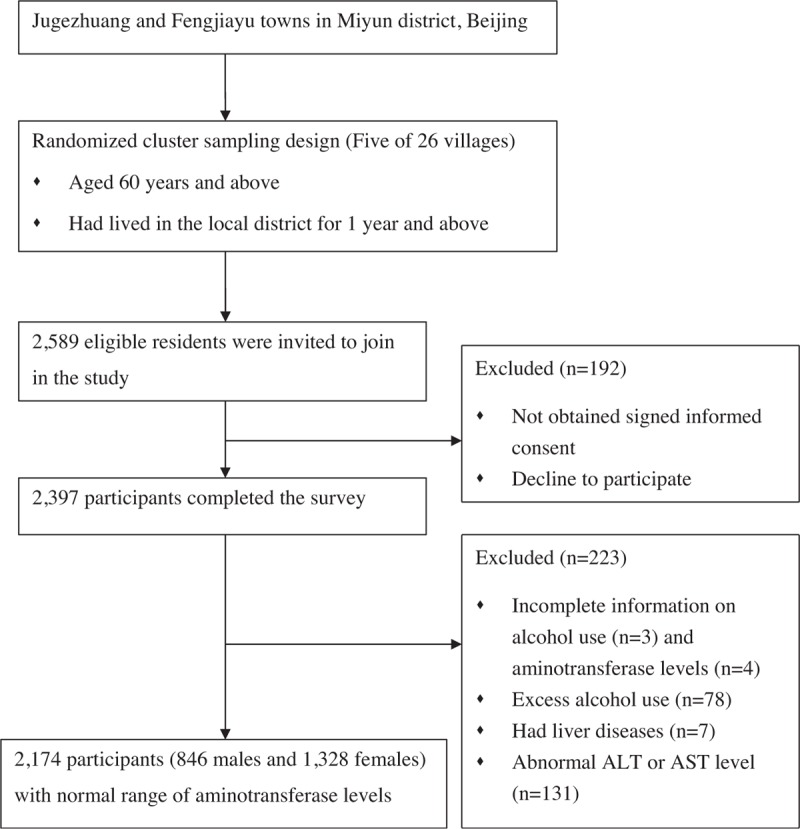
Flow diagram of the study participants.

Detailed study design has been published in our previous study.^[17]^ In brief, a cross-sectional study was conducted in Jugezhuang and Fengjiayu towns of Miyun district from May to September 2014. These places are representative of the geographic and economic characteristics of Chinese rural. Residents were eligible to select following the standards: aged 60 years and above; had lived in the local district for 1 year and above. Of the selected 26 villages, a total of 2589 eligible residents were invited to join in the study. Finally, 2397 participants completed the survey (response rate: 92.6%). Signed informed consent was obtained from each participant. Information on the project objectives (data were used only for scientific research) measurement, and data collection methods were reported on a form. We further excluded the participants with unknown alcohol use (n = 3) or missing ALT or AST information (n = 4). Participants with excess alcohol consumption (>20 g/d for females and >30 g/d for males, n = 78) or self-reported liver diseases (n = 7) were also excluded to avoid the presence of alcoholic liver disease. To eliminate individuals with potential liver pathology, an additional 131 participants with abnormal ALT or AST level (>35.0 IU/L in females and >40.0 IU/L in males) were excluded.^[14]^ Finally, 2174 participants (846 males and 1328 females) with normal range of aminotransferase levels were included in the present study. Independent Ethics Committee of the Chinese People's Liberation Army General Hospital (EC0411-2001) approved the present project. Figure 1 shows the detailed flow diagram of the study participants.

### 2.2 Data collection and measurement

All participants completed a face-to-face interview with a standardized questionnaire. Socio-demographic factors (age, gender, educational level, marital status, etc.), medical history, family history of hypertension, and lifestyle behaviors (physical activity, use of tobacco and alcohol, etc.) were collected by trained nurses.

Levels of education were collected based on self-reported years of formal schooling. Education levels were categorized as ≤6 years (primary school and below, including 0–6 years of formal schooling) and >7 years (primary school and above, including more than 7 years of formal schooling). Physical activity was measured based on the self-reported total minutes of physical activity per day.

The height, weight, and blood pressure of each participant were measured by trained nurses according to a standardized protocol. Height and weight were measured after removing shoes and heavy clothes. Waist circumference (WC) was measured midway between the lower rib margin and iliac crest while participants were in the standing position. Body mass index (BMI) was calculated as weight (kilograms) divided by the height (squared in meters). Cigarette smoking was defined as having smoked ≥1 cigarette per day for ≥1 year.^[18]^ Alcohol consumption was defined as drinking alcohol ≥12 times during the past year.^[19]^ Central obesity was defined as WC ≥85 cm for males and ≥80 cm for females.^[20]^ Overnight fasting blood specimens were obtained from measure of serum lipids and glucose, and the samples were sent to the central certified laboratory of Chinese People's Liberation Army general hospital within <30 minutes.

### 2.3 Measurement of aminotransferase levels and definitions

AST and ALT levels were measured on fasting morning serum samples using the Kinetic method. We analyzed the data separately for male and female participants because of their different baseline characteristics and prevalence of hypertension. The gender-specific tertiles of ALT at the normal range were ≤13.64, 13.65 to 18.00, and 18.01 to 40.00 IU/L in males, and ≤13.57, 13.58 to 17.87, and 17.88 to 35.00 IU/L in females; tertiles of AST were ≤20.43, 20.44 to 24.42, and 20.43 to 40.00 IU/L in males, and ≤19.51, 19.52 to 22.99, and 23.00 to 35.00 IU/L in females.

### 2.4 Blood pressure measurement and definitions

All participants were informed to avoid the use of cigarette, alcohol, coffee, or tea prior to the survey. Two blood pressure measurements were obtained from the right arm of each participant using a standardized mercury sphygmomanometer. If the difference between the 2 recordings was more than 5 mm Hg, the third measurement was recorded. The final measurement was recorded as the average value of the last 2 measurements. Hypertension was defined as systolic blood pressure ≥140 mm Hg and/or diastolic blood pressure ≥90 mm Hg and/or receiving hypertension treatment in the previous 2 weeks.^[21]^

### 2.5 Statistical analysis

Data were entered using Epidata software (3.1). All analyses were performed using SPSS software (SPSS Inc., Chicago, IL) for Windows (19.0). A 2-sided *P* value of <.05 was considered statistically significant.

Descriptive data were described as mean ± standard deviation or percentage for continuous or categorical variables. *T* and Chi-squared tests were used to assess the significance of difference between means and proportions. Multiple logistic regression analysis was used to estimate the association between gender-specific aminotransferase levels and the prevalence of hypertension. We calculated the unadjusted and adjusted odds ratios (ORs) and corresponding 95% confidence intervals (CIs) of continuous and tertiles for aminotransferase levels in the prevalence of hypertension.

## 3 Results

Baseline characteristics of study participants with normal range of aminotransferase levels are presented in Table 1. The mean age of the 2174 participants were 69.5 ± 6.8 years, ranged between 60 and 95 years. The female participants had a significantly higher prevalence of hypertension (56.1%) compared with the males (44.9%). The average values of ALT and AST were 16.9 ± 5.9 and 22.9 ± 4.7 IU/L in males and 16.6 ± 5.5 and 21.6 ± 4.0 IU/L in females. Male participants had a significantly higher level of AST than the females.

**Table 1:**
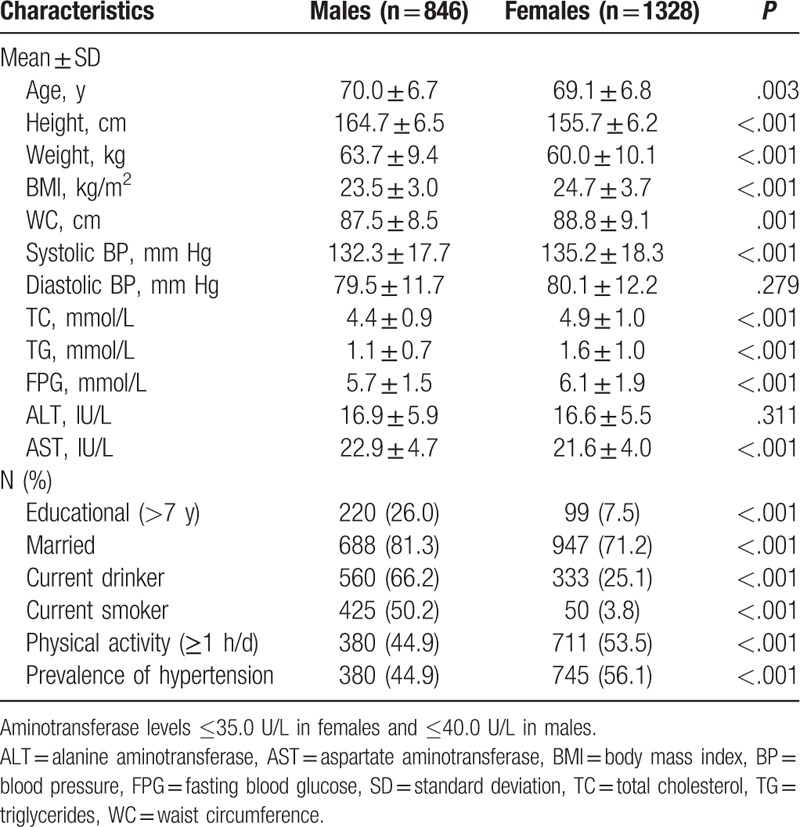
Baseline characteristics of study participants with aminotransferase levels at the normal range.

### 3.1 Association between aminotransferase levels and hypertension

Table 2 presents the OR (95% CI) of hypertension per 1 IU/L elevation of aminotransferase levels at the normal range. AST was not significantly associated with hypertension both in men and women. ALT was significantly associated with hypertension: for each 1 IU/L elevation of ALT level, the adjusted OR (95% CI) of hypertension was 1.05 (1.03, 1.07) in women and 1.03 (1.00, 1.05) in men, after adjusting for age. However, the association was no longer significant in men after adjusting for age, educational level, marital status, alcohol and cigarette use, physical activity, and family history of hypertension (model 2), or adjusted as described above plus BMI, total cholesterol, and fasting plasma glucose (model 3); but the associations remained significantly in women, and the adjusted ORs (95% CIs) of models 2 and 3 were 1.06 (1.03, 1.08) and 1.04 (1.01, 1.07), respectively.

**Table 2:**
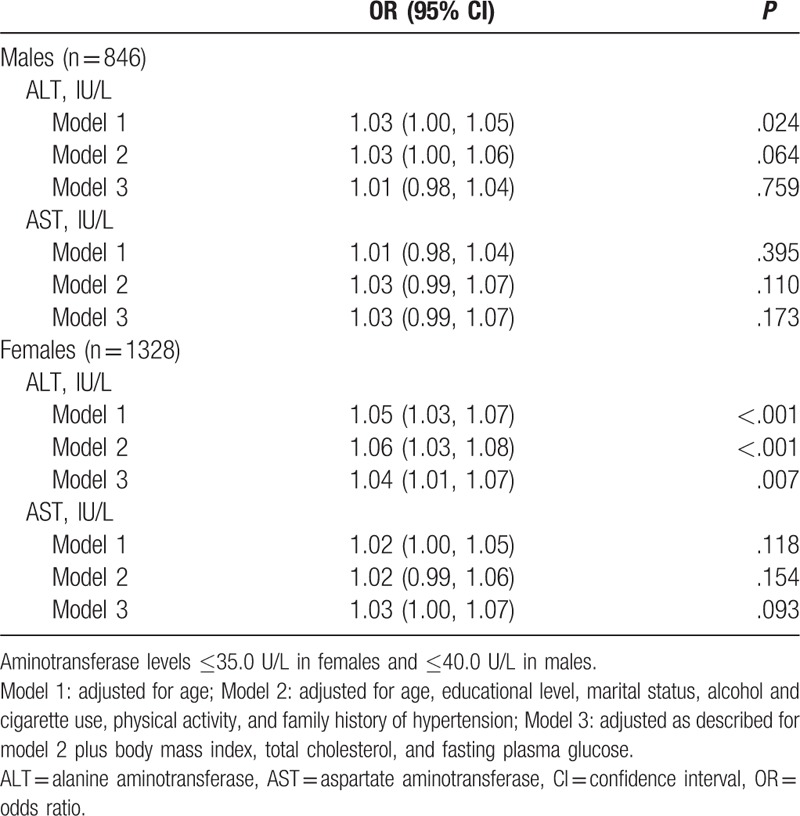
OR (95% CI) of hypertension per 1 IU/L elevation of aminotransferase levels at the normal range.

Table 3 presents the OR (95% CI) of hypertension by tertiles of aminotransferase levels at the normal range. Similar to Table 2, tertiles of AST were not significantly associated with hypertension both in men and women. There were some evidence of significant association between the highest tertile of ALT and hypertension in men, and the adjusted ORs (95% CIs) of models 1 and 2 were 1.45 (1.03, 2.04) and 1.57 (1.05, 2.33), respectively. The ORs of hypertension increased across tertiles of ALT in women, and the ORs (95% CIs) were 1.00, 1.17 (0.85, 1.60), and 1.63 (1.15, 2.31) (*P* value for trend = .021), after adjusting for confounding factors (model 3).

**Table 3:**
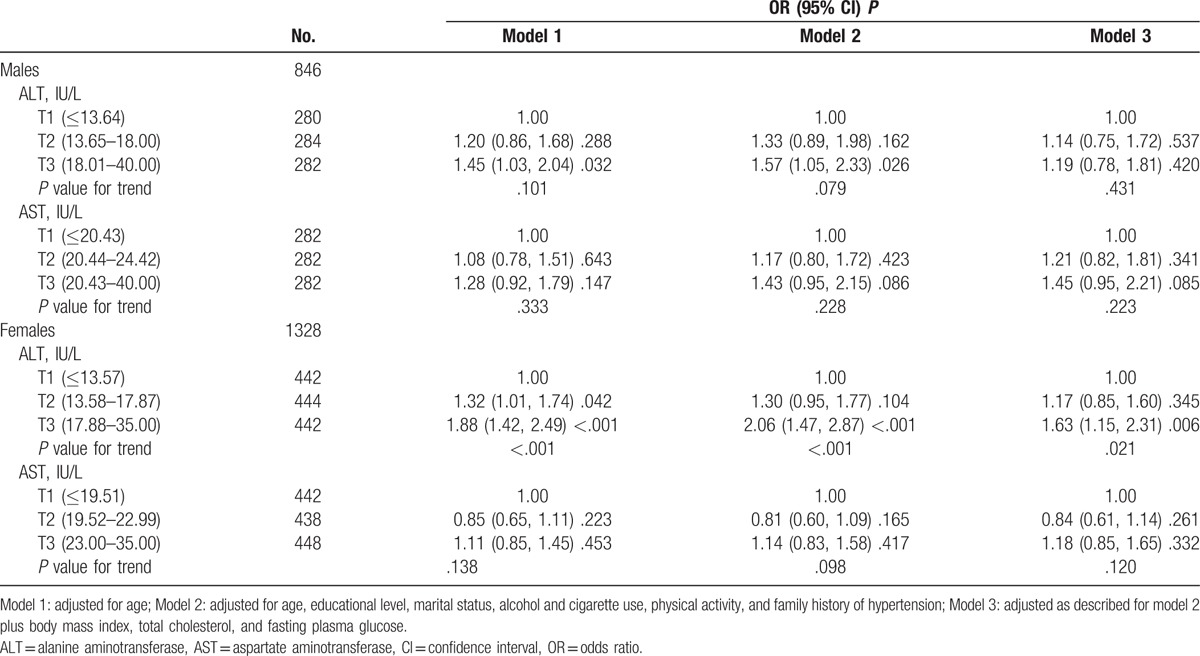
OR (95% CI) of hypertension by tertile of aminotransferase levels at the normal range.

### 3.2 Impact of other risk factors on the association

To examine the possible confounding effects, we evaluated the association between hypertension and tertiles of ALT levels by the presence of other risk factors. Table 4 shows that the association was only significant in the women with central obesity, and the ORs (95% CIs) of hypertension increased across tertiles of ALT from 1.00, 1.40 (1.04, 1.89) to 1.87 (1.38, 2.54) (*P* value for trend = .021) after adjusting for confounding factors (model 3). Table 5 shows that the association was remain significant in the nondrinking women, and the ORs (95% CIs) of hypertension increased across tertiles of ALT from 1.00, 1.31 (0.90, 1.91) to 1.71 (1.15, 2.56) (*P* value for trend = .032) (model 3). In female drinkers, the association was borderline significant in the highest tertile of ALT, and the adjusted OR (95% CI) of model 3 was 1.77 (0.99, 3.15).

**Table 4:**
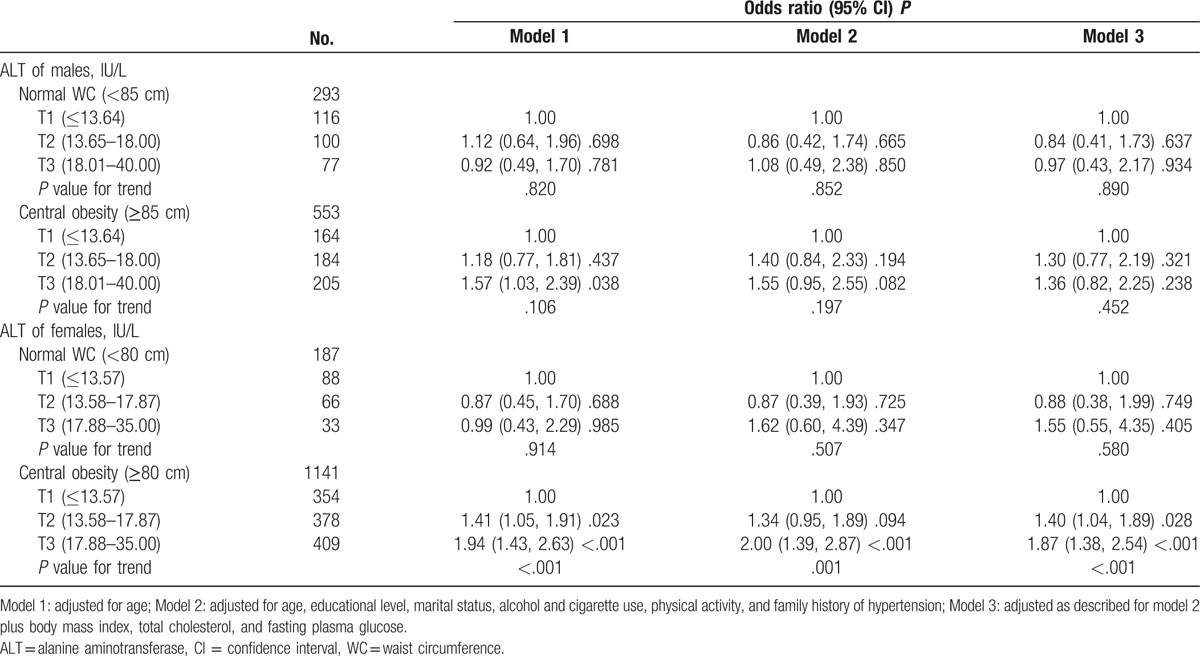
Odds ratio (95% CI) of hypertension by tertile of ALT levels at the normal range by central obesity.

**Table 5:**
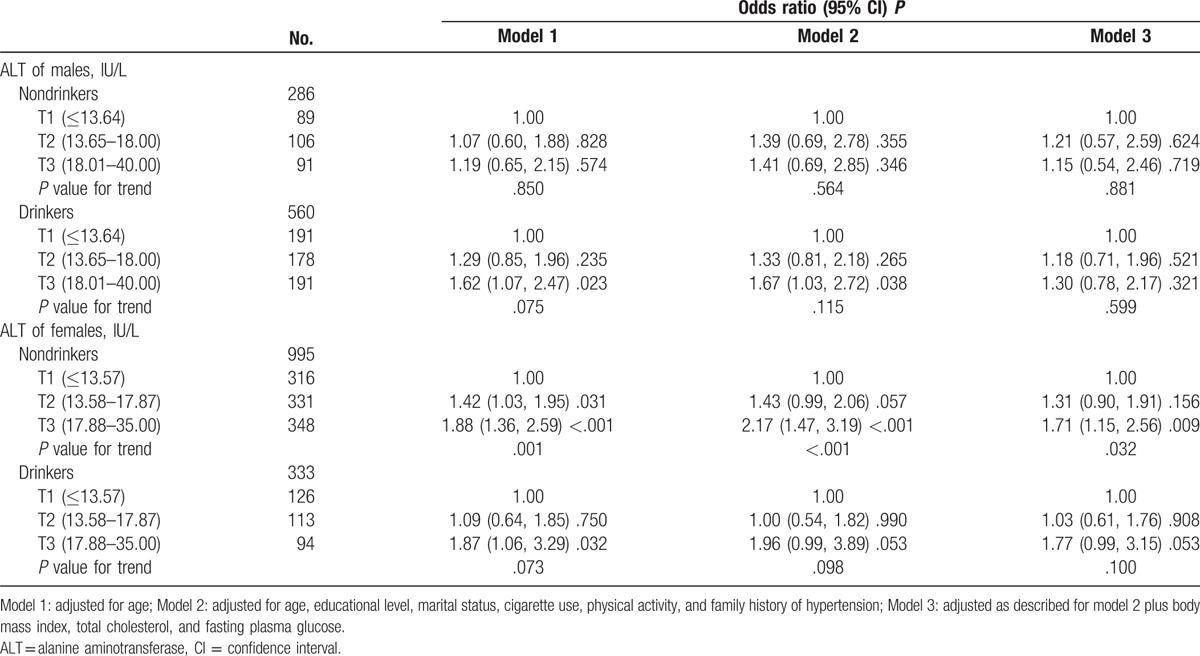
Odds ratios of hypertension by tertile of ALT levels at the normal range by current alcohol use.

## 4 Discussion

In a Chinese rural elderly population, increased serum ALT but not AST level was independently associated with hypertension, but this association was only significant in women. Previous epidemiological studies examined the association have yielded inconsistent findings. Our study supported several previous studies which reported the positive association of increased ALT but not AST level.^[14–16]^ In addition, we provided evidence for the association in the normal range of ALT level among the Chinese elderly people, which indicated that even mild ALT elevation as a sensitive indicator for hypertension. ALT is a specific marker of liver pathology and a strong biomarker for hepatic insulin sensitivity and liver fat accumulation; however, AST is less liver-specific, and it is released by damage to the liver, and also tissues and organs.^[22]^ Significant associations between ALT level and the development of diabetes, stroke, and vascular disease have been previously reported.^[23–25]^ Considering the highly comorbidity between hypertension and those chronic diseases, the above findings may partly support our finding.^[23–25]^ The mechanism of the association is not fully understood and still required to be studied in the future.

We observed a gender difference in the relationship of ALT level and hypertension. One possible explanation of the gender difference is that the causes of ALT elevation may differ by sex. For example, if other factor is a main cause of elevated ALT in men but not in women, the relationship may be different by sex. In addition, the negative finding might be due to low statistical power. The proportion of men with excessive alcohol consumption was large, and excessive drinkers were excluded from the analysis sample; as a result, the total number of male participants was relatively small. In fact, we observed an evidence of significant association in men, but the association was nonsignificant after adjusting for confounding factors. The association of ALT level and hypertension may be dependent of other confounding factors in men. Additional studies are needed to explore the potential gender difference of the association.

Further analysis revealed that the effect of elevated ALT on hypertension was only significant in women with increased central fat distribution. This indicates that obesity enhances the effect of ALT, and fatty liver may represent an important underlying mechanism for this association. Moreover, the association between hepatic insulin sensitivity and fatty liver has been shown in several clinical studies.^[26]^ As a stronger biomarker of hepatic insulin sensitivity, it is mechanically supported the association between ALT level and hypertension. Furthermore, we found that the association was existed in nondrinkers, which indicated that the association was not solely caused by alcohol consumption. The above finding is in accordance with previous study which reported that ALT is an epidemiological marker of NAFLD.^[8]^ Borderline significant association was observed in the relationship of ALT level with hypertension in women drinkers, we conjectured that the small number of female drinkers (about 100 participants in each subgroup) limited us to observe the potential association.

Limitations of the present study should be illustrated. First, because of the cross-sectional design, causal relationship cannot be established. Second, elevations of serum aminotransferase levels are not specific, other diseases, such as hepatitis, chronic disease, and biliary diseases can also involved elevated aminotransferase levels. Third, blood pressure and aminotransferase levels were measured only in a single day, and thus possible misclassification might have been given. Fourth, some important risk factors were relied on self-report, measurement bias cannot be avoided. For instance, alcohol consumption was linked with both aminotransferase levels and elevated blood pressure, a measurement error in alcohol use might lead to residual confounding. Finally, we cannot control other unmeasured confounding variables in the analysis, and the absence of potential confounders may have contributed to our findings. Prospective studies are required to evaluate the role of elevated aminotransferase levels in the development of hypertension. Experimental studies are needed to support the epidemiological evidence and to explore the biological mechanisms underlying the possible gender-related association.

## 5 Conclusions

In conclusion, ALT level was significantly associated with hypertension only in women in a Chinese rural elderly population. Further studies are warranted to explore the possible gender-related association and to extend them to different populations.
